# Association of Nonalcoholic Fatty Liver Disease with Subclinical Cardiovascular Changes: A Systematic Review and Meta-Analysis

**DOI:** 10.1155/2015/213737

**Published:** 2015-07-26

**Authors:** Enea Bonci, Claudio Chiesa, Paolo Versacci, Caterina Anania, Lucia Silvestri, Lucia Pacifico

**Affiliations:** ^1^Department of Experimental Medicine, Sapienza University of Rome, 00161 Rome, Italy; ^2^Institute of Translational Pharmacology, National Research Council, 00133 Rome, Italy; ^3^Department of Pediatrics and Child Neuropsychiatry, Sapienza University of Rome, 00161 Rome, Italy

## Abstract

In the last 20 years, nonalcoholic fatty liver disease (NAFLD) has become the leading cause of chronic liver disease worldwide, primarily as a result of the epidemic of obesity. NAFLD is strongly associated with insulin resistance, glucose intolerance, and dyslipidemia and is currently regarded as the liver manifestation of the metabolic syndrome, a highly atherogenic condition even at a very early age. Patients with NAFLD including pediatric subjects have a higher prevalence of subclinical atherosclerosis, as shown by impaired flow-mediated vasodilation, increased carotid artery intima-media thickness, and arterial stiffness, which are independent of obesity and other established risk factors. More recent work has identified NAFLD as a risk factor not only for premature coronary heart disease and cardiovascular events, but also for early subclinical abnormalities in myocardial structure and function. Thus, we conducted a systematic review and meta-analysis to test the hypothesis that NAFLD is associated with evidence of subclinical cardiac structural and functional abnormalities.

## 1. Introduction

In the last 20 years, nonalcoholic fatty liver disease (NAFLD) has become the leading cause of chronic liver disease worldwide, primarily as a result of the epidemic of obesity [1–4]. NAFLD is a spectrum of fat-associated liver conditions that can result in end-stage liver disease and the need for liver transplantation [5–7]. Simple steatosis, or fatty liver, occurs early in NAFLD and may progress to nonalcoholic steatohepatitis (NASH), fibrosis, and cirrhosis with increased risk of hepatocellular carcinoma [5–7]. NAFLD is strongly associated with obesity, insulin resistance, hypertension, and dyslipidemia and is now regarded as the liver manifestation of the metabolic syndrome (MetS) [8–10], a highly atherogenic condition even at a very early age [11–13]. When compared to control subjects who do not have hepatic steatosis, patients with NAFLD have a higher prevalence of atherosclerosis, as shown by increased carotid wall intimal thickness, increased numbers of atherosclerotic plaques, and increased plasma markers of endothelial dysfunction, which are independent of obesity and other established risk factors [13–19]. Consistent with these observations, natural history studies have reported that the increased age-related mortality observed in patients with NAFLD is attributable to cardiovascular as well as liver-related deaths [11, 20]. More recent work has identified NAFLD as a risk factor also for early subclinical abnormalities in myocardial metabolism as well as in cardiac structure and function [21–29]. In particular, it has been shown that NAFLD is associated with myocardial insulin resistance, altered cardiac energy metabolism, left ventricular (LV) hypertrophy, and impaired diastolic function [23–29]. However, a comprehensive evaluation of the impact of NAFLD on these complications is lacking. Thus, we conducted a systematic review and meta-analysis to test the hypothesis that NAFLD is associated with evidence of subclinical cardiac structural and functional abnormalities.

## 2. Methods

### 2.1. Search Strategy

A systematic literature search was conducted by two researchers (LP and CA) to identify all articles (published from January 2000 to September 2014) that assessed by echocardiography cardiac geometry and function in NAFLD patients. We performed the search in Medline, EMBASE, and the Cochrane Library. Search terms were NAFLD OR NASH OR nonalcoholic fatty liver disease OR nonalcoholic steatohepatitis OR fatty liver OR liver fat OR steatosis OR liver enzymes OR transaminase OR ALT OR AST OR GGT OR severity of liver disease AND left ventricle OR left ventricular hypertrophy OR cardiac hypertrophy OR cardiac dysfunction OR ventricular dysfunction OR echocardiography. Search results were limited to English language publications. References of included articles were manually searched for other relevant studies.

### 2.2. Study Selection

Inclusion criteria were observational studies including quantitative data on LV structure and/or function assessed by echocardiography and NAFLD diagnosed by (1) liver histology, (2) imaging (ultrasound, computed tomography, magnetic resonance imaging (MRI), or spectroscopy (MRS)), or (3) biochemistry (elevations in serum aspartate aminotransferase, alanine aminotransferase (ALT), or gamma-glutamyl transferase). Competing causes of steatosis, including alcohol consumption and viral, autoimmune, and metabolic hepatitis, had to be excluded. Only the updated or largest report was considered when multiple publications by the same research group were found.

### 2.3. Data Extraction and Quality Assessment

Data extraction was performed independently by two authors (EB and LP) and included title, authors, date of publication, study design, inclusion and exclusion criteria used in the study, number of patients and controls, the diagnostic procedures for NAFLD, the echocardiographic methods, and LV structural and functional measures. All quantitative echocardiographic variables had to be expressed as means ± SD; otherwise SE or 95% confidence intervals (CI) were used to estimate the SD.

The quality of the selected studies was assessed independently by two authors (EB and CC) using the Newcastle-Ottawa Scale (NOS) for cohort and cross-sectional studies. The NOS uses a “star” rating system to judge quality based on three aspects of the study: selection of study groups, comparability of study groups, and ascertainment of either the exposure or outcome of interest [36]. Any discrepancies were addressed by a joint reevaluation of the original article with another author.

### 2.4. Statistical Analysis

We calculated effect size for the following measures of LV structure and function: LV mass index, early mitral velocity (*E*)/late mitral velocity (*A*) ratio, and *E*/early diastolic tissue velocity (*e*′) ratio. We used the standardized difference, which is the mean difference (between cases and controls) divided by the common within-group standard deviation for cardiac parameters. We chose the standardized mean difference as a measure of association instead of the weighted mean difference since the outcome variables had been determined by different instruments. Fixed or random-effect models were used to summarize results. The standardized differences were evaluated using the Der-Simonian and Laird procedures. In this analysis, the variation among studies is incorporated and thus the overall outcome estimates have greater standard errors. The Der-Simonian and Laird method uses weights equal to the reciprocal of the variance of the individual outcome estimates and thus takes account of the variation between them. Heterogeneity was estimated using the *I* ^2^ index. If the *I* ^2^ value was <50%, a fixed-effect meta-analysis was applied. If the *I* ^2^ value was ≥50%, a random-effect meta-analysis was used.

We used Cohen's categories to evaluate the magnitude of the effect size, calculated by the standardized mean difference, where *d* of 0.2–0.5 indicates a small effect size, *d* > 0.5–0.8 indicates a medium effect size, and *d* > 0.8 indicates a large effect size [37].

Publication bias was checked visually by creating a funnel plot with the standard error plotted against the mean difference of single LV parameters. In addition, publication bias was statistically tested by Egger's test. Consistent with recommendations to determine stability in the meta-analytic results, we computed the classic fail-safe *N* analysis [38]. This technique calculates the number of studies with nonsignificant effects required to nullify the overall effect determined in the current analysis. Larger fail-safe *N* values increase confidence in the overall effect and validate the stability of the current findings. Data were analyzed with the use of the Comprehensive Meta-Analysis software, version 2 (Biostat, Englewood, New Jersey).

## 3. Results

### 3.1. Characteristics of the Studies

**Figure 1:**
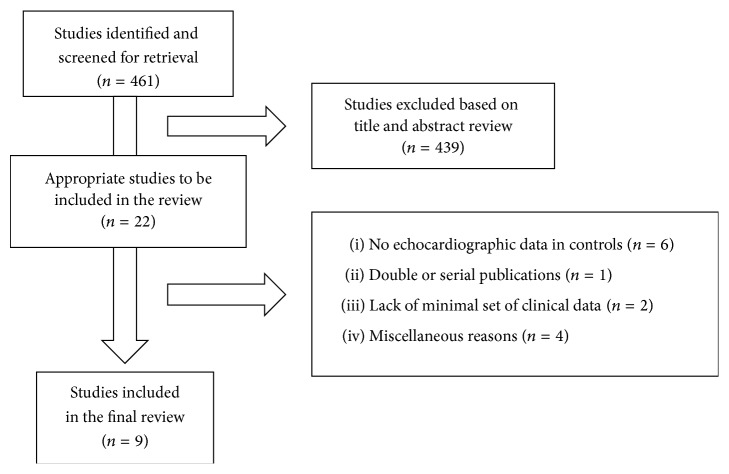
Schematic flow chart for the selection of studies.

The first literature search identified 461 papers; 22 of these were potentially eligible for the analysis, but only 9 studies, six involving adults [25, 26, 30–33] and three with children and adolescents [29, 34, 35], could be included in the final review (Figure 1). Overall the methodological quality of the selected studies was satisfactory.

#### 3.1.1. Adult Studies

Table 1(a) summarizes the studies on the effects of NAFLD on cardiac structure and function in adults. Overall, 629 adult patients with NAFLD and 1237 controls were included in the studies. Control subjects were matched for age and sex in some studies and for age, sex, and body mass index (BMI) or for age, sex, BMI, and waist circumference in others. Five studies were hospital-based case-control ones [25, 26, 30–32], and one was a population-based cross-sectional survey [33]. In all cases and controls, LV geometry and function was measured by M-mode and pulsed Doppler echocardiography; tissue Doppler imaging (TDI) was also used in the majority of studies (5 of the 6 studies) (Table 1).

NAFLD was defined by liver histology in one study, by ultrasound in four studies, and by computed tomography scan in one study. Three studies enrolled exclusively nondiabetic, normotensive individuals, one study enrolled type 2 diabetic patients, and one study enrolled never-treated essential hypertensive patients. The population-based study included patients with NAFLD and MetS.

#### 3.1.2. Pediatric Studies

Table 1(b) summarizes the studies on the effects of NAFLD on cardiac structure and function in children and adolescents [29, 34, 35]. Overall, 244 obese children with NAFLD, 680 obese subjects without liver involvement, and 236 healthy controls matched for age and gender were included in the studies. In all cases and controls, LV geometry and function was measured by M-mode, pulsed Doppler, and TDI.

NAFLD was defined by ultrasound in one study, by ultrasound and elevated serum ALT in one study, and by MRI (and liver biopsy in a subgroup) in one study.

### 3.2. Cardiac Parameters

The parameters reported across the selected publications varied among studies, but LV mass indexed to height^2.7^ or to body surface area (BSA), *E*/*A* ratio, the early annular diastolic tissue velocity (*e*′), and *E*/*e*′ ratio were the most frequently reported outcomes.

#### 3.2.1. Adult Studies

**Figure 2:**
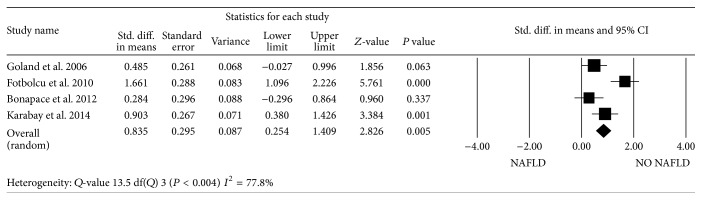
Forest plots show the comparison of LV mass indexed to BSA between NAFLD patients and NO NAFLD subjects.

**Figure 3:**
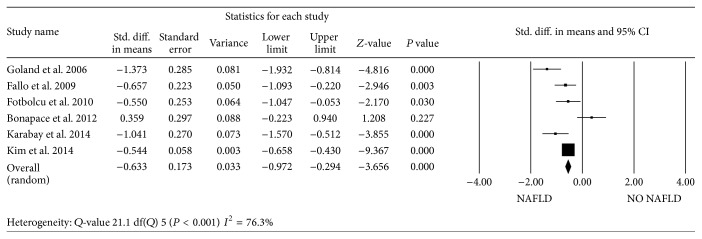
Forest plots show the comparison of early mitral velocity (*E*)/late mitral velocity (*A*) ratio between NAFLD patients and NO NAFLD subjects.

**Figure 4:**
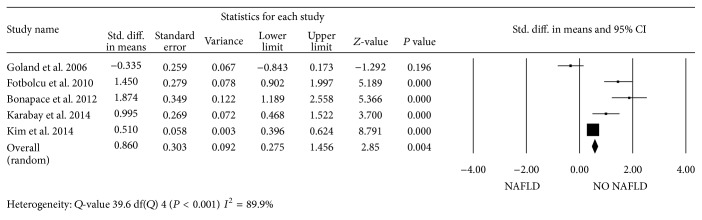
Forest plots show the comparison of *E*/early diastolic tissue velocity (*e*′) ratio between NAFLD patients and NO NAFLD subjects.

Two studies reported LV mass indexed to height^2.7^ [30, 33] and 4 studies the LV mass indexed to BSA [25, 26, 31, 32]. Four studies found an increased LV mass index in NAFLD patients compared to controls. When pooling the data on LV mass indexed to height^2.7^ as well as those on LV mass indexed to BSA, the standardized difference in means was statistically significant (*P* < 0.0001) only for LV mass indexed to BSA (Figure 2). There was significant evidence of heterogeneity among the studies. *E*/*A* ratio was available in all 6 studies. On average, a worse *E*/*A* ratio was found in NAFLD patients, with a standardized mean difference of −0.57 (95% CI, −0.67 to −0.47; *P* < 0.0001) (Figure 3). *E*/*e*′ ratio was reported in 5 of the 6 studies. Patients with NAFLD had higher *E*/*e*′ ratio than those without NAFLD in 4 of the 5 studies, leading to a standardized mean difference of 0.56 (95% CI, 0.45 to 0.66; *P* < 0.0001) (Figure 4). Again, there was heterogeneity among studies.

We found no evidence of publication bias based on Egger's test with respect to *E*/*A* ratio (intercept = −0.55 (95% CI, −4.55 to 3.46); *P* = 0.72) and *E*/*e*′ ratio (intercept = 1.95 (95% CI, −5.01 to 8.90); *P* = 0.44). The classic fail-safe *N* was relatively high to lower the significance of this meta-analysis (number of missing studies that would make *P* value greater than alpha = 120).

#### 3.2.2. Pediatric Studies

**Figure 5:**
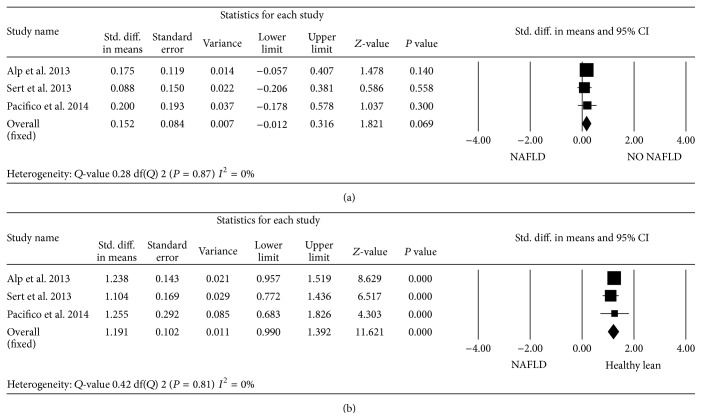
Forest plots show the comparison of LV mass indexed to height^2.7^ between NAFLD and NO NAFLD obese children and adolescents (a) and between obese children with NAFLD and healthy lean subjects (b).

**Figure 6:**
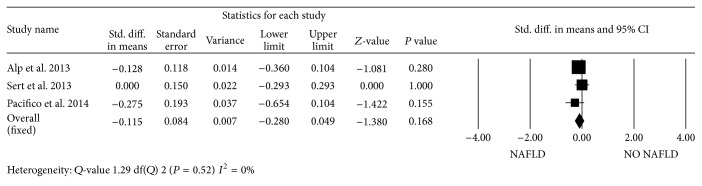
Forest plots show the comparison of early mitral velocity (*E*)/late mitral velocity (*A*) ratio between NAFLD and NO NAFLD obese children and adolescents.

**Figure 7:**
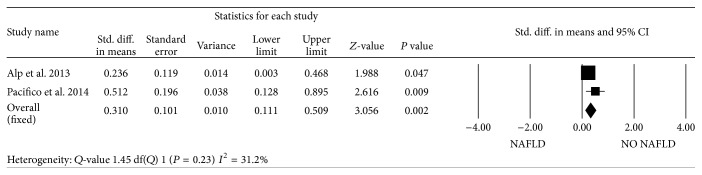
Forest plots show the comparison of *E*/early diastolic tissue velocity (*e*′) ratio between NAFLD and NO NAFLD obese children and adolescents.

When pooling the data on LV mass indexed to height^2.7^, the standardized mean difference in LV mass between obese children with NAFLD and obese subjects without liver involvement did not reach statistical significance (*P* = 0.069). In contrast, when compared to healthy lean controls, the standardized mean difference in LV mass was statistically significant (mean, 1.19 (95% CI, 0.99 to 1.39); *P* < 0.0001) (Figure 5). In all studies, no significant differences were found in *E*/*A* ratio between the groups (Figure 6). However, obese children and adolescents with NAFLD had higher *E*/*e*′ ratio than those without NAFLD, leading to a standardized mean difference of 0.31 (95% CI, 0.11 to 0.51; *P* < 0.001) (Figure 7). In contrast to adult population, there was no evidence of heterogeneity among pediatric studies. We found no evidence of publication bias based on Egger's test with respect to *E*/*A* ratio (intercept = −1.41 (95% CI, −4.46 to 3.76); *P* = 0.72). Visual inspection of the funnel plot suggested the absence of publication bias for the *E*/*e*′ ratio, which was identified in 2 of the 3 eligible studies. However, the classic fail-safe *N* was relatively low when considering obese children with and without NAFLD, suggesting that only tenuous conclusions should be drawn.

## 4. Discussion

While previous systematic reviews and meta-analyses have investigated the association between NAFLD and the risk of cardiovascular events (such as myocardial infarction, angina pectoris, and ischemic stroke), cerebrovascular disease (such as cerebral hemorrhage), and peripheral vascular disease [39], or the effect of current treatments on NAFLD-associated cardiometabolic conditions [40], in the present systematic review and meta-analysis, the association of subclinical cardiac structure and function abnormalities in patients with NAFLD has been examined.

We found that NAFLD patients both adults and children have increased features of diastolic LV dysfunction. In fact, NAFLD adult patients had a lower *E*/*A* ratio and a higher *E*/*e*′ ratio, while children with NAFLD had higher *E*/*e*′ ratio.

Diastolic LV dysfunction is increasingly acknowledged as a major contributor to the development of heart failure, but as the early onset of heart failure with preserved systolic function (e.g., preclinical diastolic dysfunction) is asymptomatic, it is often not diagnosed in the early stages [41]. Although systolic function is well characterized by determinations of ejection fraction, diastolic function characterization of the heart's stiffness, relaxation, and pressure changes is more difficult. Invasive measures of rate of LV pressure decline, LV relaxation time constant, and stiffness modulus can characterize diastolic function [42]. Echocardiography, a common noninvasive imaging technique, is useful in determining the presence of systolic or diastolic dysfunction. In the diastole, the LV filling pattern consists of 2 phases: early and late atrial contraction. The *E*/*A* ratio is used as an estimate of the relaxation pattern of the ventricle. Furthermore, TDI can be used to measure myocardial motion, specifically the rate at which the mitral annulus moves toward the base during early diastole (*e*′). The LV filling pressures can be estimated by the *E*/*e*′ ratio [42]. Thus *E*/*A* ratio and *E*/*e*′ ratio are reliable markers of LV diastolic function.

In addition to LV diastolic dysfunction, our meta-analysis showed significant differences in LV mass index, a continuous measure of cardiac structure, between adult patients with NAFLD and controls. In the pediatric population, the standardized mean difference in LV mass between obese children with NAFLD and obese subjects without liver involvement did not reach statistical significance. However, when compared to healthy lean controls, the standardized mean difference in LV mass was statistically significant. Although LV diastolic dysfunction is known to be associated with the development of LV hypertrophy, investigations in both human and animal models of hypertension suggest that early LV diastolic dysfunction may precede the development of LV hypertrophy [43].

It should be pointed out that, in a meta-analysis, the presence of heterogeneity in study design and clinical characteristics of subjects may influence the interpretation of the pooled risk estimates. However, even using the random-effects model, taking account of the variation between the different studies yielded a statistically significant estimate of LV dysfunction. Of note, there was no evidence of statistically significant heterogeneity among the studies involving children.

The severity of liver disease was not considered in most of the studies. This may be due to liver biopsy being invasive and expensive and the ever present possibility of complications. Only one study in adults and one in children disclosed cardiac data according to liver histology and showed conflicting results. Karabay et al. [32] found no significant differences in cardiac dysfunction among NAFLD groups (e.g., simple steatosis, borderline NASH, and definite NASH). In contrast, Pacifico et al. [29] showed that obese children with NAFLD have features of early LV dysfunction, compared to obese children without NAFLD and lean controls. Notably, when the group of obese subjects was divided according to the presence of NASH, it was evident that some functional cardiac differences were more pronounced in the group of NASH. Therefore, some misclassification of individuals with NASH as controls would probably tend to reduce the strength of the association between NAFLD and cardiac abnormalities. Interpretation of the results of this meta-analysis is limited by other caveats; that is, a small number of studies met the inclusion criteria, and all the reported analyses relied on the standardized mean differences between patients and controls, without adjustment for other potential confounder factors.

Although the pathogenesis of cardiac dysfunction in NAFLD is still unclear, insulin resistance, abnormal lipid profile, and low-grade inflammatory state have been suggested to play a role [44–47]. Hepatic steatosis is associated with hepatic insulin resistance, which means that hepatic glucose production is impaired, leading to hyperglycemia and compensatory hyperinsulinemia. This may worsen both systemic and cardiac insulin resistance. The liver plays an important role in controlling the amount of circulating lipids. In patients with NAFLD, the increase in free fatty acids may lead to myocardial lipid accumulation, with consequent alterations in LV function [46–48]. In fact, myocardial steatosis may cause changes in myocardial substrate metabolism and efficiency (as measured by cardiac work/myocardial oxygen consumption) that occur early in the process leading to impaired LV contractility [44, 45]. Using ^1^H-MRS, Rijzewijk et al. showed that the intramyocardial fat content was significantly higher in uncomplicated type 2 diabetic men than in nondiabetic controls and was related to impaired cardiac metabolism [49]. Epicardial adipose tissue (EAT), being metabolically active, produces proatherogenic, proinflammatory, and prothrombotic adipocytokines [50, 51]. Its anatomic location, without any barrier to the adjacent myocardium, results in local paracrine interaction between EAT and the myocardium [52]. Perseghin et al. who used cardiac MRI and ^31^P-MRS showed that patients with fatty liver had increased epicardial fat and abnormal cardiac metabolism [24]. Thus, epicardial and myocardial fat represent abnormal ectopic fat storage and may be a marker of the cumulative effects of NAFLD and insulin resistance in the setting of pathological adiposity [48, 52], leading to cardiovascular complications [53].

More recently, it has been shown that the liver activates homeostatic mechanisms which increase the production and export of non-HDL-C to reduce the toxic effect of excess cholesterol due to diet. Thus, the liver may limit free cholesterol toxicity, but a consequence is that APOB-containing atherogenic lipoproteins are produced, which may, at least in part, account for the increased cardiovascular risk in patients with NAFLD [54]. Emerging evidence also suggests that NAFLD, especially in its necroinflammatory form, might be involved in the pathogenesis of cardiac function abnormalities through the systemic release of several mediators from the steatotic and inflamed liver (including C-reactive protein, interleukin-6, tumor necrosis factor-*α*, and other proinflammatory cytokines) [11, 55].

Genetic studies have highlighted several single nucleotide polymorphisms that may characterize patients with a high risk for NAFLD development and progression [56–60]. In particular, a common missense variant, rs738409 (I148M), in the patatin-like phospholipase 3 (*PNPLA3*) gene has been associated not only with increased hepatic fat content and serum liver enzymes but also with increased risk of NASH and fibrosis progression [56, 58]. Recently, genetic variation in the transmembrane 6 superfamily member 2 protein (TM6SF2) at rs58542926 has been shown to confer susceptibility to NAFLD, independent of genetic variation in PNPLA3 at rs738409 [59, 60]. Of note, the E167K variant in TM6SF2 is associated with a distinct subtype of NAFLD, characterized by preserved insulin sensitivity with regard to lipolysis, hepatic glucose production, and lack of hypertriglyceridemia despite a clearly increased liver fat content [61]. In addition, Dongiovanni et al. [62] found that the TM6SF2 E167K variant increases susceptibility to NASH and liver fibrosis but protects against cardiovascular disease (CVD). Their findings suggest that inhibition of secretion of very-low-density lipoproteins from the liver protects against CVD, but at the cost of an increased risk of severe liver disease.

In conclusion, our analysis supports the association between NAFLD and subclinical cardiovascular changes. However, confirmation in large cross-sectional and less heterogeneous studies is needed. If LV dysfunction in NAFLD is confirmed, future research directions should include strategies and interventions to prevent cardiac disease progression in patients with NAFLD.

## Figures and Tables

**(a):** 

Author/ country/ year [reference]	Study design, population, and sample size	Diagnosis	Outcomes	Main results	Comment	NOS score
Goland et al./ Israel/ 2006 [25]	Cross-sectional. Nondiabetic, normotensive patients with NAFLD (*n* = 38) and age- and sex-matched healthy controls (*n* = 25). NAFLD patients included those with metabolic syndrome.	Liver ultrasound and liver biopsy in a subgroup of 11 NAFLD patients.	LV structure and function (M-mode echocardiography; pulsed and tissue Doppler echocardiography).	Patients with NAFLD had mild changes in cardiac geometry (thickening of the interventricular septum and posterior wall and increased LV mass) as well as significant differences in parameters of diastolic function compared with the control group.	All data were adjusted for BMI. On multivariate regression analysis, including all the metabolic and echocardiographic variables, *e*′ on TDI was the only independent parameter associated with NAFLD.	7

Fallo et al./ Italy/ 2009 [30]	Cross-sectional. Never-treated essential hypertensive patients with (*n* = 48) or without (*n* = 38) fatty liver. The 2 groups were similar as to sex, age, and blood pressure levels.	Liver ultrasound.	LV structure and function (M-mode echocardiography and pulsed Doppler echocardiography).	NAFLD patients had similar prevalence of LV hypertrophy compared to subjects without NAFLD, but a higher prevalence of LV diastolic dysfunction.	Multivariate logistic regression analysis showed that HOMA-IR and diastolic dysfunction remained independently associated with NAFLD.	7

Fotbolcu et al./ Turkey/ 2010 [26]	Cross-sectional. Nondiabetic, normotensive patients with NAFLD (*n* = 35) and control subjects (*n* = 30). The 2 groups were similar as to sex and age.	Liver ultrasound.	LV structure and function (M-mode echocardiography and pulsed and tissue Doppler echocardiography).	Patients with NAFLD had changes in cardiac geometry (thickening of the interventricular septum and posterior wall and increased LV mass) as well as significant differences in parameters of systolic and diastolic function compared with the control group.	No correlation was found between BMI and waist circumference and *e*′ and *s*′ on TDI.	7

Bonapace et al./ Italy/ 2012 [31]	Cross-sectional. T2DM patients with (*n* = 32) and without (*n* = 18) fatty liver. The 2 groups were similar as to sex, age, BMI, waist circumference, and diabetes duration.	Liver ultrasound.	LV structure and function (M-mode echocardiography and pulsed and tissue Doppler echocardiography).	T2DM patients with fatty liver showed LV diastolic dysfunction, even if the LV morphology and systolic function were preserved.	LV dysfunction remained significant after adjustment for hypertension and other cardiometabolic risk factors.	8

Karabay et al./ Turkey/ 2014 [32]	Cross-sectional. NAFLD patients (*n* = 55)^*∗*^ and healthy controls (*n* = 21; normal laboratory values and liver ultrasound). ^*∗*^Of the 55 NAFLD patients, 9 had simple steatosis, 24 borderline NASH, and 22 definite NASH.	Liver biopsy.	LV structure and function (M-mode echocardiography, pulsed and tissue Doppler echocardiography, and speckle tracking echocardiography).	Patients with NAFLD and its subgroups had changes in cardiac geometry (thickening of the interventricular septum and posterior wall and increased LV mass) as well as significant differences in parameters of diastolic function compared with the control group.	Speckle tracking echocardiography showed no differences in strain between subgroup patients (simple steatosis versus borderline NASH versus definite NASH).	7

Kim et al./ Korea/ 2014 [33]	Population-based. Cohort included in the Korean Genome and Epidemiology study: NAFLD patients without MetS (*n* = 180); NAFLD patients with MetS (*n* = 241); NO NAFLD without MetS (*n* = 1105); NO NAFLD with MetS (*n* = 360).	Liver computed tomography.	LV structure and function (M-mode echocardiography and pulsed and tissue Doppler echocardiography).	Compared with subjects with neither NAFLD nor MetS, those with both disorders showed the most significant differences in structural and functional LV parameters, such as LV mass index, transmitral Doppler indices, and systolic/diastolic TDI values.	In multivariate analyses, NAFLD and MetS were each significantly and independently associated with TDI *e*′ velocity.	7

T2DM: type 2 diabetes mellitus; BMI: body mass index; HOMA-IR: homeostasis model assessment of insulin resistance; MetS: metabolic syndrome; NAFLD: nonalcoholic fatty liver disease; LV: left ventricular; NASH: nonalcoholic steatohepatitis; NOS: Newcastle-Ottawa Scale; TDI: tissue Doppler imaging.

**(b):** 

Authors/ country/ year [reference]	Study design, population, and sample size	Diagnosis	Outcomes	Main results	Comments	NOS score
Alp et al./ Turkey/ 2013 [34]	Cross-sectional. Obese children and adolescents with (*n* = 93)^*∗*^ and without (*n* = 307) NAFLD matched for gender and age and control subjects (*n* = 150). ^*∗*^Of the 93 NAFLD children, 67 had ultrasonographic grade 1 steatosis and 26 had grade 2 steatosis.	Liver ultrasound.	LV structure and function; epicardial fat (M-mode echocardiography and pulsed and tissue Doppler echocardiography).	Increased end-systolic thickness of the interventricular septum and larger LV mass, as well as LV systolic and diastolic dysfunction, were found in NAFLD group. In addition, obese children with NAFLD had increased epicardial fat thickness.	On logistic regression analysis including anthropometric and metabolic variables, total adipose tissue mass percentage and IVSs were the only independent parameters associated with liver steatosis.	8

Sert et al./ Turkey/ 2013 [35]	Cross-sectional. Obese adolescents with (*n* = 97) and without (*n* = 83) NAFLD and control subjects (*n* = 68).	Liver ultrasound and elevated serum alanine aminotransferase.	LV structure and function (M-mode echocardiography and pulsed and tissue Doppler echocardiography).	Obese adolescents with NAFLD exhibited increased LV dimensions and mass, as well as LV diastolic dysfunction.		7

Pacifico et al./ Italy/ 2014 [29]	Cross-sectional. Obese children and adolescents with (*n* = 54) and without (*n* = 54) NAFLD matched for age, gender, Tanner stage, and BMI-SD score and healthy control subjects (*n* = 18) matched for gender, age, and pubertal status.	Hepatic magnetic resonance imaging and liver biopsy in a subgroup of 41 NAFLD patients (26 had definite NASH and 15 were not-NASH).	LV structure and function; epicardial fat (M-mode echocardiography and pulsed and tissue Doppler echocardiography).	Increased interventricular septum thickness at end-diastole and at end-systole, as well as LV systolic and diastolic dysfunction, was found in NAFLD group. Children with more severe liver histology had worse LV dysfunction than those with more mild liver changes. NAFLD group had also increased epicardial fat thickness.	Patients with definite NASH had significantly lower *e*′ velocity and significantly higher *E*-to-*e*′ and Tei index (*P* < 0.001, resp.) than those without NASH. In multiple logistic regression analysis, NAFLD was the only statistically significant variable associated with increased *E*-to-*e*′ ratio, whereas NAFLD and systolic blood pressure were significantly associated with increased Tei index.	8

NAFLD: nonalcoholic fatty liver disease; LV: left ventricular; BMI: body mass index; NASH: nonalcoholic steatohepatitis; NOS: Newcastle-Ottawa Scale.

## Abbreviations

*A*: Late mitral velocity
ALT: Alanine aminotransferase
BMI: Body mass index
BSA: Body surface area
CI: Confidence intervals
*E*: Early mitral velocity
*e*′: Early diastolic tissue velocity
EAT: Epicardial adipose tissue
LV: Left ventricular
MetS: Metabolic syndrome
MRI: Magnetic resonance imaging
MRS: Magnetic resonance spectroscopy
NAFLD: Nonalcoholic fatty liver disease
NASH: Nonalcoholic steatohepatitis
NOS: Newcastle-Ottawa Scale
TDI: Tissue Doppler imaging.
